# An Entangled Model for Sustainability Indicators

**DOI:** 10.1371/journal.pone.0135250

**Published:** 2015-08-21

**Authors:** Pável Vázquez, Jesús A. del Río, Karla G. Cedano, Manuel Martínez, Henrik J. Jensen

**Affiliations:** 1 Instituto de Investigación en Ciencias Básicas y Aplicadas, Universidad Autónoma del Estado de Morelos, Cuernavaca, Morelos, México; 2 Instituto de Energías Renovables y Centro de Ciencias de la Complejidad, Universidad Nacional Autónoma de México, Temixco, Morelos, México; 3 InnoBa, Cuernavaca, Morelos, México; 4 Instituto de Energías Renovables, Universidad Nacional Autónoma de México, Temixco, Morelos, México; 5 Department of Mathematics and Center for Complexity Science, Imperial College London, London, United Kingdom; Potsdam Institute for Climate Impact Research, GERMANY

## Abstract

Nowadays the challenge for humanity is to find pathways towards sustainable development. Decision makers require a set of sustainability indicators to know if the sustainability strategies are following those pathways. There are more than one hundred sustainability indicators but they differ on their relative importance according to the size of the locality and change on time. The resources needed to follow these sustainability indicators are scarce and in some instances finite, especially in smaller regions. Therefore strategies to select set of these indicators are useful for decision makers responsible for monitoring sustainability. In this paper we propose a model for the identification and selection of a set of sustainability indicators that adequately represents human systems. In developing this model, we applied evolutionary dynamics in a space where sustainability indicators are fundamental entities interconnected by an interaction matrix. we used a fixed interaction that simulates the current context for the city of Cuernavaca, México as an example. We were able to identify and define relevant sets indicators for the system by using the Pareto principle. In this case we identified a set of sixteen sustainability indicators with more than 80% of the total strength. This set presents resilience to perturbations. For the Tangled Nature framework we provided a manner of treating different contexts (i.e., cities, counties, states, regions, countries, continents or the whole planet), dealing with small dimensions. This model provides decision makers with a valuable tool to select sustainability indicators set for towns, cities, regions, countries, continents or the entire planet according to a coevolutionary framework. The social legitimacy can arise from the fact that each individual indicator must be selected from those that are most important for the subject community.

## Introduction

The concept of sustainability provides solutions to human needs and basic desires. Sustainable development “seeks to meet the needs and aspirations of the present without compromising the ability to meet those of the future” [1]. It means that whatever we do now should not harm future generations. This approach entails so many aspects that sustainable development must address the problems from a holistic point of view; accordingly, sustainability requires that different aspects be tackled simultaneously.

**Fig 1:**
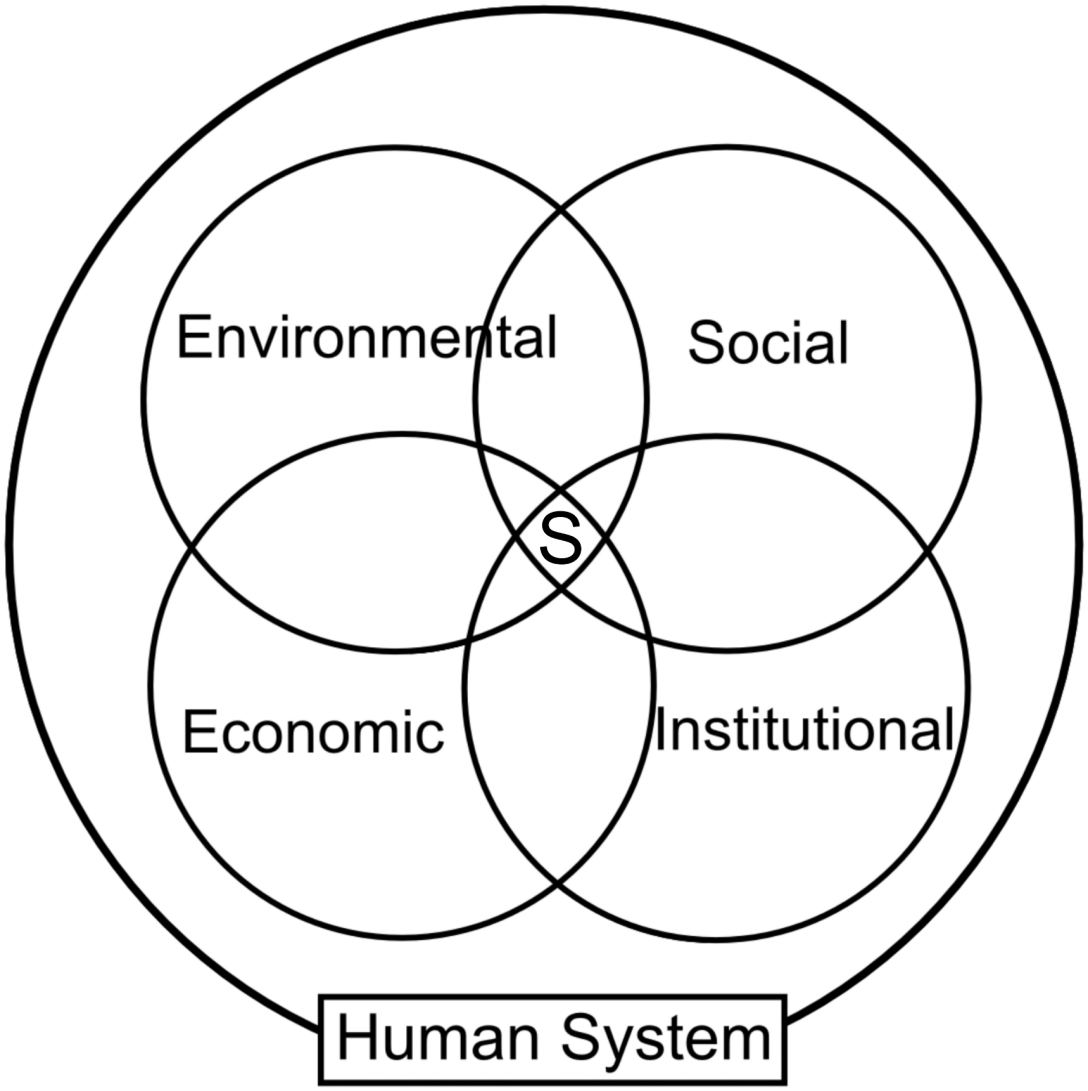
Dimensions of sustainability. The intersection of the four elements is the sustainability.

Sustainability has four components: economic, environmental, social and institutional. These components are called dimensions, usually represented as pillars using a Venn diagram with four partially overlapped circles as shown in Fig 1, where the intersection of the four components is considered to represent the sustainable. Fig 1 represents the conceptual linking of the four sustainability dimensions and their indicators among themselves. This provides insight into grouping indicators as multidimensional areas. That is, indicators do not belong exclusively to a single dimension, rather they are a composite of the four dimensions.

Stresses from each dimension are interlinked, sustainability dimensions can complement or oppose in competitive relations. An environmental example is deforestation, increasing deforestation accelerates soil erosion and siltation of rivers and lakes. Another example is energy consumption for industrialization. This energy consumption is associated with the global greenhouse effect that in turn acidifies and land [1]. These and many other examples must be studied in real-life situations, so decision makers must be aware if an objective is being achieved or not. Sustainability indicators (SIs) are used for that matter.

Sustainability indicators (SIs) are used for these studies. SIs measure different dimensions of sustainablity, however because dimensions are linked, indicators are also interrelated. For instance, water quality is a SI that measures the environmental dimension, but is also and indicator that is strongly related to social and institutional dimensions.

Also not all SIs are useful in specific contexts and not all SIs can be applied in different regions. For example, the Marine Trophic Index indicator can be measured only in coastal regions. Another consideration are the resources needed to achieve the measure, which are usually limited. SIs are also subject to multiple pressures, agendas and trends. Selecting the most relevant SIs is usually done by experts, using their knowledge and expertise to decide which indicators should be used. With the aim of incorporating the richness and complexity of human relationships and continuing a holistic approach, we have constructed a model in this work that addresses the problem of finding the suitable sustainability indicators set for a specific context viewed from the perspective of complex systems.

The specific context used depends on spatial scale and time factors, primarily because economic, social and institutional situations and ecological conditions differ widely among places. Accordingly, each location has to develop and define its own concrete policy. Spatial scale refers to farms, communities, countries or even the whole world. In many cases boundaries are ambiguous. For instance, a city is not usually, if ever, entirely self sustained, because food is typically supplied from many different places outside city boundaries. Thus, sustainability of cities it is heavily influenced by surroundings. Another complicating factor pertaining to context is political boundaries, which are subject to controlling laws and constitutions. However, even when boundaries are in fact porous, typical theoretical perspective treats specific locations as closed systems, meaning there is no interaction with the outside. According to the World Commission on Environment and Development (WCED) the sustainability definition time scale starts with present generations, however number of future generations is relative and the number of how many generations should be considered into sustainability is not ill-defined. Another concept can be used for that matter, many authors have defined sustainable development as the situation where the quality of the system increases or keeps unchanged over time [2]. This quality provides the context in which the process takes place and is commonly considered the life quality, defined as *An individual’s perception of their position in life, in the context of the culture and values in which they live and in relation to their goals, expectations, standards and concerns* [3].

The sustainability definition by itself implies a complex and diverse system that should be approached with a holistic point of view [4, 5]. In the next section we consider sustainability as a complex system, so it shares properties suitable to be modeled using a biological coevolution framework. We will define the Entangled Sustainability Model and its properties, and thereafter we will give some implications of the behavior of the model and the results obtained from simulating a specific region.

Sustainability refers to human systems, implicitly necessitating defining it as a complex system. We already mentioned that SIs are linked one to another in a system of interconnected components so it has two components: *elements* and *connections* [6]. In our model these elements are the sustainability indicators and the connections are the relations between the economy, the society, the environment and the institutions. Meanwhile human systems are evolutionary, open and highly unpredictable. Complex systems addresses systems in terms of the environment in which the universe is seen as a dynamic network of interrelated events, in which none of the properties of any part of the network is fundamental. The whole dynamics are derived from the properties of the parts and the overall consistency of their interrelations determines the structure of the entire network [7].

The Entangled Sustainability Model features unique properties: the existence of mechanisms of co-evolution, where elements receive and give answers to their own behaviours and to other elements using a feedback that drives the system with self organized way. These mechanism cause the emergence of patterns and structures at a higher level. This emergence phenomenon can be considered an emerging adaptation, but not all complex systems are adaptive and not all adaptations increase the survival of the system [8]. When structure arises to improve the “effectiveness” of the whole, this system is considered to be a Complex Adaptive System (CAS) [9–11]. In the Entangled Sustainability Model we propose that sustainability components evolve together. These elements are the SIs, and their behaviour is reflected in the interactions between sustainability dimensions. This means we are using two levels of interaction, the first one is between dimensions (seen as the context) and the other one is between individual indicators in which dynamics occur.

In our model environment does not evolve simultaneously with SIs, but is implicit in the interactions between dimensions. These dimensional relations are used to test the model. Applying a multi-methodological approach, we assessed the relations by surveying experts on how the relations between dimensions should be in a specific context. We use the average of this survey. As said before, the model does not include any interaction with external events, meaning that boundaries are fixed and not permeable.

On the other hand, only allowing intrinsic interaction does not ensure a sustainable system. According to the definition of sustainability, a quantity must exist that increases or remains constant over time. We call this quantity the fortitude *F*(*t*)^*α*^, a dynamic value inherit to each indicator *α*, representing the importance or the relevance it has in the system.

## Methodology

Indicators are the main tool to determine the health of the system and to know if objectives are being achieved. However for economic reasons decision makers and politicians prioritize attention to only a few indicators to optimize the goal of resource management. Keeping this goal in mind, our model explores the problem of finding the optimal indicators set without requiring the expert to decide the appropriate level of indicators. Rather, the only condition is defining the system’s universe, where indicators co-evolve interconnectedly through a correlated interaction matrix. The interaction matrix for indicators is derived from a simpler interaction matrix between sustainability dimensions used as a particular context. For this purpose we have decided to base our model on the Entangled Economy Model [12], a model inspired by evolutionary ecology [13–15]. In ecology an ecosystem emerges from cooperation and competition dynamics between biological agents that co-exist in an environment. In a similar way, in sociology a sustainable situation can emerge from a co-evolving interaction between its own social beings, each agent dependent on outputs from the other. Similar to an ecological system, our model mimics ecology as the co-evolution of societal components in a static closed environment. The Tangled Nature model provides the framework where species compete for resources in the physical environment and populations increase or diminish. In a similar manner, the Entangled Economy model describes the behavior of companies using the capital as fortitude. In our model, sustainability indicators cooperate and compete for properly describing the pathway to sustainability. Efforts to relate sustainability and organisms have been made [16] and different ways to model sustainability can be found in the literature [17], the model proposed here is the first of its kind to view indicators as coevolutionary, similar as in an ecological system. The model explores situations in which indicators compete during simulation steps, so it considers a sustainable situation where a long stable situation arises. During this stable situation a number of indicators are able to capture the entire dynamics even when a perturbation is applied, this property is the resilience of the entangled sustainability model. Sustainability involve four dimensions, in our model each indicator is a vector in a four dimensional space, denoted by $I^{\alpha }=(I_{1}^{\alpha },\;I_{2}^{\alpha },\;I_{3}^{\alpha },\;I_{4}^{\alpha })=(\text{Environmental},\text{Economic},\text{Social},\text{Institutional})$. Additionally, each indicator is a representation of the four sustainability dimensions; that is, each measures a different aspect that is important for sustainability. These values have different affinities to the different dimensions of sustainability. Thus possible indicators symbolize agents, for example an agent represented by the vector (3,0,0,0), which has only a strong environmental component, while (3,3,3,3) represents an indicator in which all the components are the strongest. Since it is possible that multiple sustainability indicators fit the same vector, by construction we can use consensus and voting criteria for the specific vector representation. If two indicators are represented by the same vector then the two indicators are actually. By contrast, a small difference between two indicators it is represented as a small deviation in the four dimensional space. For instance, the emission of greenhouse gases indicator is strongly related to the environmental dimension but also to the economic [18], so this indicator would be represented in the model as a vector similar to (2,2,0,0).

An agent *α* has a total fortitude *F* ^*α*^(*t*) at time *t*. We used *F*_*α*_ ∈ [0, 3] producing a space of *N* = *L* ^4^ = 256 total indicators. Every indicator is then identified by a label in the range 1 to 256.

Two mechanisms define the level of involvement. The first mechanism is spatial competition. Two close indicators in space have similar sustainability components, meaning that two close indicators represent similar measures of sustainability and therefore compete for fortitude. The level of competition between two indicators decays exponentially and is defined by:

$$C\left(\alpha ,\beta \right)=\exp[\frac{\left(-1/4\right)\Delta I_{i}^{\alpha \beta }}{\xi }]\;$$ (1)

with $\Delta I_{i}^{\alpha \beta }=|\sum_{i=1}^{4}b_{i}(I_{i}^{\alpha }-I_{i}^{\beta })|$, the distance between indicators.

The second interaction is a cooperation, in which an indicator *α* is coupled with another *β* with a value *J*(*α*, *β*) ≠ *J*(*β*, *α*).

Because the context defines the interaction between dimensions, we used this information to create the interaction between indicators *J*(*α*, *β*). The matrix *J* ^0^ of size 4 × 4 represents relations in the dimensions level and it is composed like Eq (2).

$$J^{0}\;=\;\begin{array}{cc} & \begin{array}{cccc} En & Ec & So & In \end{array}\\ \begin{array}{c} En\\ Ec\\ So\\ In \end{array} & \left(\begin{array}{cccc} 0 & En\to Ec & En\to So & En\to In\\ Ec\to En & 0 & Ec\to So & Ec\to In\\ So\to En & So\to Ec & 0 & So\to In\\ In\to En & In\to Ec & In\to So & 0 \end{array}\right) \end{array}$$ (2)

Values can be chosen between [−*c*, *c*] and self-interaction is deemed to be neutral (null). For example, interaction of an environmental dimension and an economic is indicated as *En* → *Ec*. An impairment is a negative number, while a positive is a contribution and zero is neutrality.*J* ^0^ then defines the context in which the problem is being considered. To represent a specific scenario *J* ^0^ is created using a survey of experts who estimate the values of interaction using an averaging procedure or consensus process (see S1 Text). This interaction matrix has sufficient flexibility such that using different values simulate different contexts.

To translate this into an indicators interaction we used the expression in Eq (3).

$$J\left(\alpha \beta \right)=\sum_{i=1}^{L}\sum_{j=1}^{L}I_{i}^{\alpha }J_{ij}^{0}I_{j}^{\beta }$$ (3)

Using *N* = 256 agents then all of the 65536 interactions are represented by *J* of size 256 × 256. This interaction matrix between indicators inherits the properties of the dimensional interaction matrix, so that the autointeraction is zero and the matrix is not symmetric.

The ability of each indicator to gain fortitude is given by a probability determined by a weight function Eq (4)

$$H\left(\alpha ,t\right)=a_{1}\frac{\sum_{\beta =1}^{N\left(t\right)}J\left(\alpha ,\beta \right)}{\sum_{\beta =1}^{N\left(t\right)}C\left(\alpha ,\beta \right)}-a_{2}\sum_{\beta =1}^{N\left(t\right)}C\left(\alpha ,\beta \right)-a_{3}\frac{N\left(t\right)}{R\left(t\right)}$$ (4)

The first term of the weight function Eq (4) determines the effect of interactions with all other indicators that have nonzero fortitude, weighted by the overall level of competition. The numerator is the sum of all values in the matrix of interaction *J*(*α*, *β*) with the indicator *α* with all of the other indicators with *F* ^*β*^(*t*) ≠ 0. To consider indicators that are in a similar sustainability sector and are thus competing for fortitude, it is normalized by the values of total competition relating to *α*.

The second term of the weight function is directly and negatively affected by the spatial competition, where the impact between two negative indicators decays exponentially.

The last term in Eq (4) regulates growth, *R*(*t*) restricts the amount of resources and *N*(*t*) is the number of active indicators at time *t*, i.e. indicators with fortitude *F* ^*α*^(*t*) ≠ 0. When *N*(*t*) increases by one unit *R*(*t*) decreases by one unit. This illustrates that active indicators needs resources. In this way the sum of both is a constant, *R*(*t*) + *N*(*t*) = *const*. If the number of indicators is small the term *a*_3_ *N*(*t*)/*R*(*t*) it is also small which encourages strong weight function, unlike a large set of indicators with nonzero strength results in a big term and thus the weight function decreases. The coefficients *a*_*i*_ are constants of similar magnitude for all three terms.

The weight function *H*(*α*, *t*) is used in ecological contexts [13–15] as the ability of one specie to reproduce and in economics the ability to create capital [12] here we use *H*(*α*, *t*) to represent the ability of one indicator to gain fortitude or relevance in the system. Since the weight function is a real value, *H*(*α*, *t*) ∈ ℝ, a probability is then calculated for each indicator associated with losing or gaining fortitude.

$$P_{g}=\frac{\exp[H(\alpha ,t)]}{1+\exp[H(\alpha ,t)]}.$$ (5)

Each indicator with nonzero fortitude is updated at time *t* according to the value of the corresponding *H*(*α*, *t*) and *P*_*g*_ ∈ [0, 1). We compare *P*_*g*_ to a random number, *u*, uniformly distributed on [0,1). If *u* < *P*_*g*_, the indicator gains fortitude, if *u* ≥ *P*_*g*_ loses fortitude.

An indicator gains fortitude *F*(*α*, *t*) as shown in Eq (6):

$$F^{\alpha }\left(t+1\right)=F^{\alpha }\left(t\right)(1+c_{g}\frac{J^{+}\left(\alpha \right)}{J_{Tot}\left(\alpha \right)}).$$ (6)

Where *J* ^+^( *alpha*) is the sum of all positive interactions of indicator *α* and *J*_*Tot*_(*α*) is the sum total in absolute value of the interactions matrix *J*, where constant *c*_*g*_ serves to control the growth. The equation for loss of fortitude is similar, with a loss constant *c*_*p*_. In this case *J* ^−^(*α*) is the sum of the absolute value of all negative interactions.

$$F^{\alpha }\left(t+1\right)=F^{\alpha }\left(t\right)(1-c_{p}\frac{J^{-}\left(\alpha \right)}{J_{Tot}\left(\alpha \right)}).$$ (7)

It is unrealistic to have indicators that entirely decouple from the dynamics. This will happen if *J* ^+^(*α*) = 0 or *J* ^−^(*α*) = 0. To avoid this situation we make replace *J* ^+^(*α*)(*α*) and *J* ^−^(*α*)) by *J*_*min*_(*α*) > 0 if the absolute value of *J* ^+^ or *J* ^−^ falls below *J*_*min*_. With probability $p_{g}^{0}=1/2$, gain and loss of fortitude is given by:

$$F^{\alpha }\left(t+1\right)=F^{\alpha }\left(t\right)(1+c_{g}\frac{J_{min}\left(\alpha \right)}{J_{Tot}\left(\alpha \right)}).$$ (8)

$$F^{\alpha }\left(t+1\right)=F^{\alpha }\left(t\right)(1-c_{p}\frac{J_{min}\left(\alpha \right)}{J_{Tot}\left(\alpha \right)}).$$ (9)

We have substituted the zero value for *J*_*min*_, the lowest interaction value for such indicator. An indicator with less strength than a threshold *U*_*k*_ is no longer relevant for participation with other indicators, so it dies and *F* ^*α*^(*t* + 1) = 0.

If the fortitude of an indicator exceeds a threshold *U*_*m*_, with constant probability *P*_*t*_ a percentage *c*_*t*_ of its fortitude transfers to a neighbor within a radius *r*. With these parameters the system is able to transfer fortitude to different areas of the space, allowing the fortitude to explore the different indicators and provide the opportunity to gain relevance over time. After having defined the space and dynamic rules, we now turn to the simulation of the coevolution of the indicators.

## Results and Discussion

In the present modelling framework a sustainable configuration is identified as a configuration in which the total fortitude increases or remains almost constant for a very long time. We first model parameters to obtain such a configuration.

First, coefficients of the weight function *a*_1_, *a*_2_ and *a*_3_ in Eq (4) were adjusted to avoid rapid fortitude gain or loss. Four parameters are responsible for the different stationarity behaviors, mutation rate *P*_*t*_, mutation radius *r*, mutation quantity *c*_*t*_ and the coupling strength *J*(*α*, *β*). The first three coefficients are related, in that they are responsible of the movement of fortitude through space, by increasing mutation rate and the quantity transferred. The fortitude is subject to intermittent dynamics in which its magnitude changes abruptly during sharp transitions. A high rate but small radius translates into smaller shifts, but a bigger radius moves fortitude far from the origin so duration of metastable states is increased.

**Fig 2:**
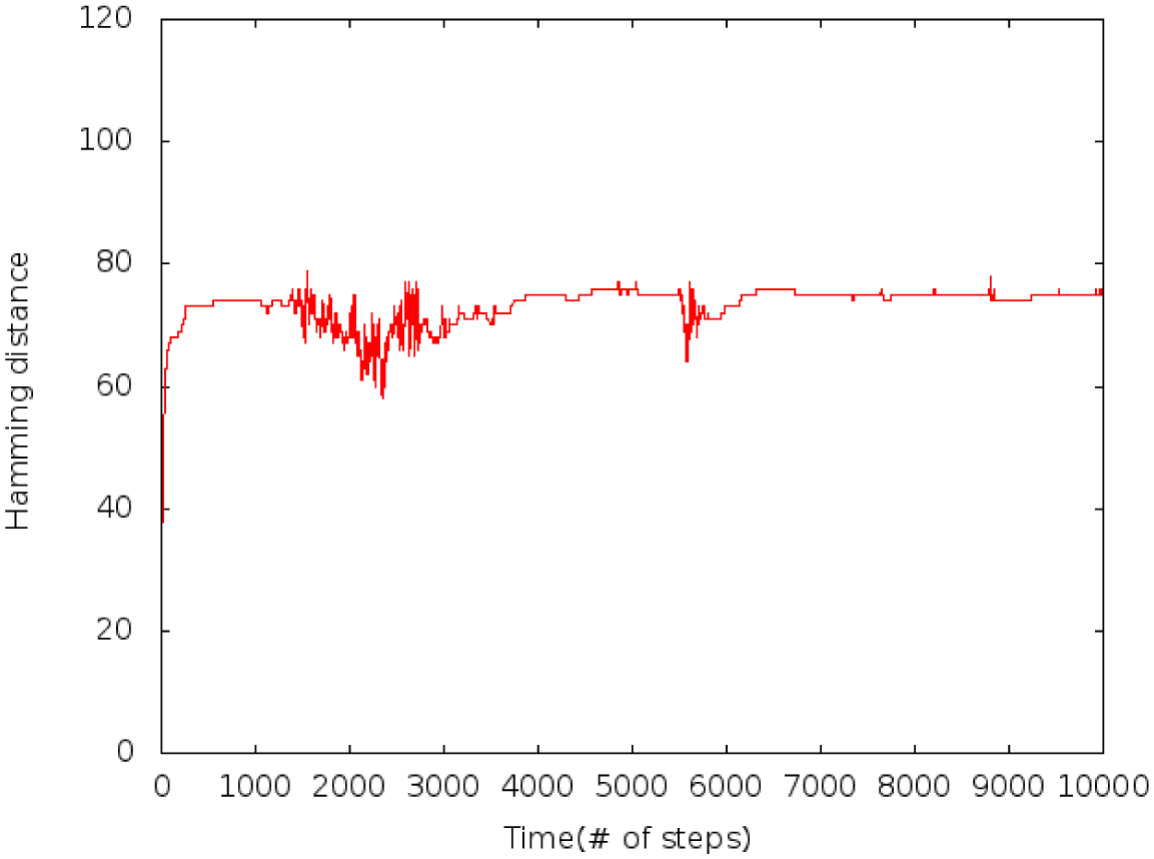
Hamming distance at time *t*. Show transitions characterized by high fluctuations. *c*_*t*_ = 0.03, *r* = 20 and *J* ∈ [−300, 300].

Using strong couplings, metastable states are observed with sudden transitions. Fig 2 shows the Hamming distance between the initial random configuration and the subsequent steps. Greater distances correspond to larger differences. Smaller couplings produce an early stationary behavior without any fortitude movement. Because we want to reproduce a human system we focused on the case with strong links where variations can happen.

**Fig 3:**
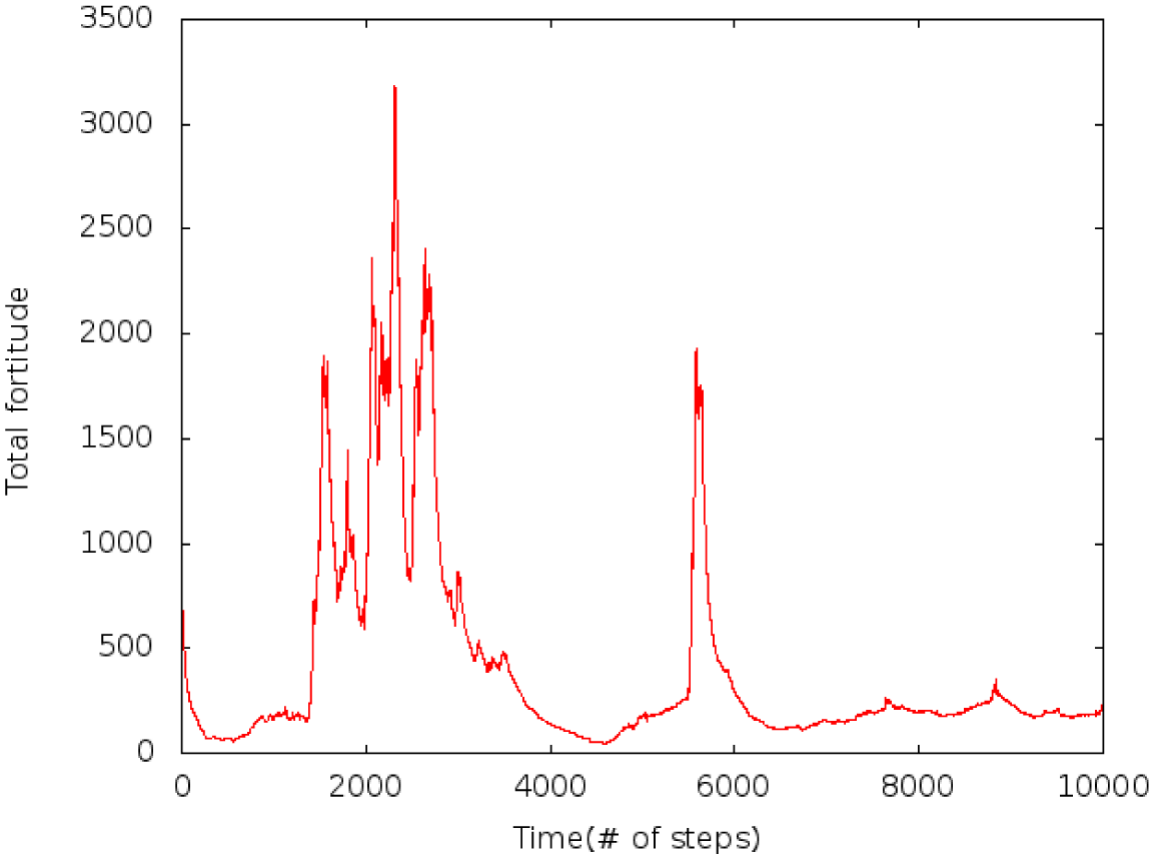
Example showing transitions and metastable states. The total fortitude is the sum of all indicators fortitude at time *t*. *c*_*t*_ = 0.03, *r* = 20 and *J* ∈ [−300, 300].

Rapid fluctuations occur during transitions. In the example given, a first fortitude movement starts around t = 1500 and a smaller one near t = 5500. Meanwhile metastable states show constant values. These transitions are related to sudden increases of total fortitude in Fig 3, separated by periods of low variations.

**Fig 4:**
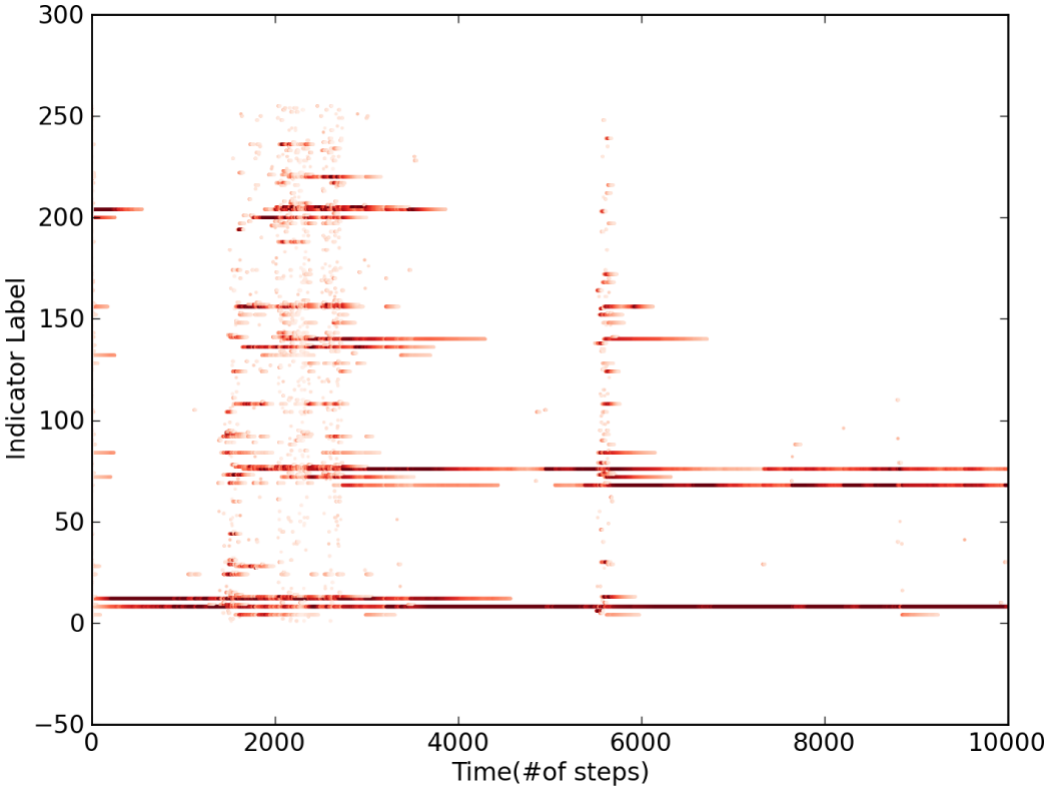
Evolution of relative fortitude by indicator. In the *y* axis indicators are labeled over time steps in the *x* axis. The higher the indicator fortitude, the darker the color representing it.

To provide an insight on how fortitude moves through the space, Fig 4 shows that must fortitude stays around a set of indicators. This configuration is interrupted by sudden rearrangement induced by transfer of fortitude to other locations and then returning to another state with the fortitude isolated on only a few active indicators.

We designed a test based on the Pareto principle to identify stable configurations, also known as the 80-20 rule, meaning that less than 20% of the population has more than 80% of the strength. The principle is asserted to appear in several different aspects of socioeconomic systems [19, 20], in our case we use this property in order to define sustainability as the set of long-lived indicators, i. e., the set of indicators that fulfill the Pareto principle and the set components does not change in time.When more than 80% of the fortitude is located in less than 20% of a group of indicators during a period of time then the system has the property of being driven by that group of indicators.

To test this, we created sets that comply with the rule. First all indicators were sorted from highest to lowest fortitude and then grouped until 80% or more of the total fortitude is added. If the number of added indicators was less than 20% (∼ 51) then it was considered a Paretian set. In Fig 4 metastable states are characterized with the same Paretian set, however transitions create rapid changes in its composition, the long lasting period where the Paretian set does not change is that in which the indicators from the set are relevant. When many rapid changes in the composition are observed, the Paretian set changes also very frequently. In those cases, the system cannot support metastable states, even after long periods.

The Paretian set during a stable configuration has small changes in its composition, so we used the union of the Paretian sets during a long lasting situation with the long state where it did not change considered to be the stable one. Using the example of Cuernavaca (Morelos, México), sixteen indicators were present in the Paretian set, for a period of 10000 steps.

$$J_{p}^{0}\;=\;\begin{array}{cc} & \begin{array}{cccc} En & Ec & So & In \end{array}\\ \begin{array}{c} En\\ Ec\\ So\\ In \end{array} & \left(\begin{array}{cccc} 0 & 0.9 & -0.3 & 0.1\\ 0.2 & 0 & -0.5 & 0.8\\ -0.3 & 0.9 & 0 & 0.1\\ 0.5 & -0.6 & -0.1 & 0 \end{array}\right) \end{array}$$

**Fig 5:**
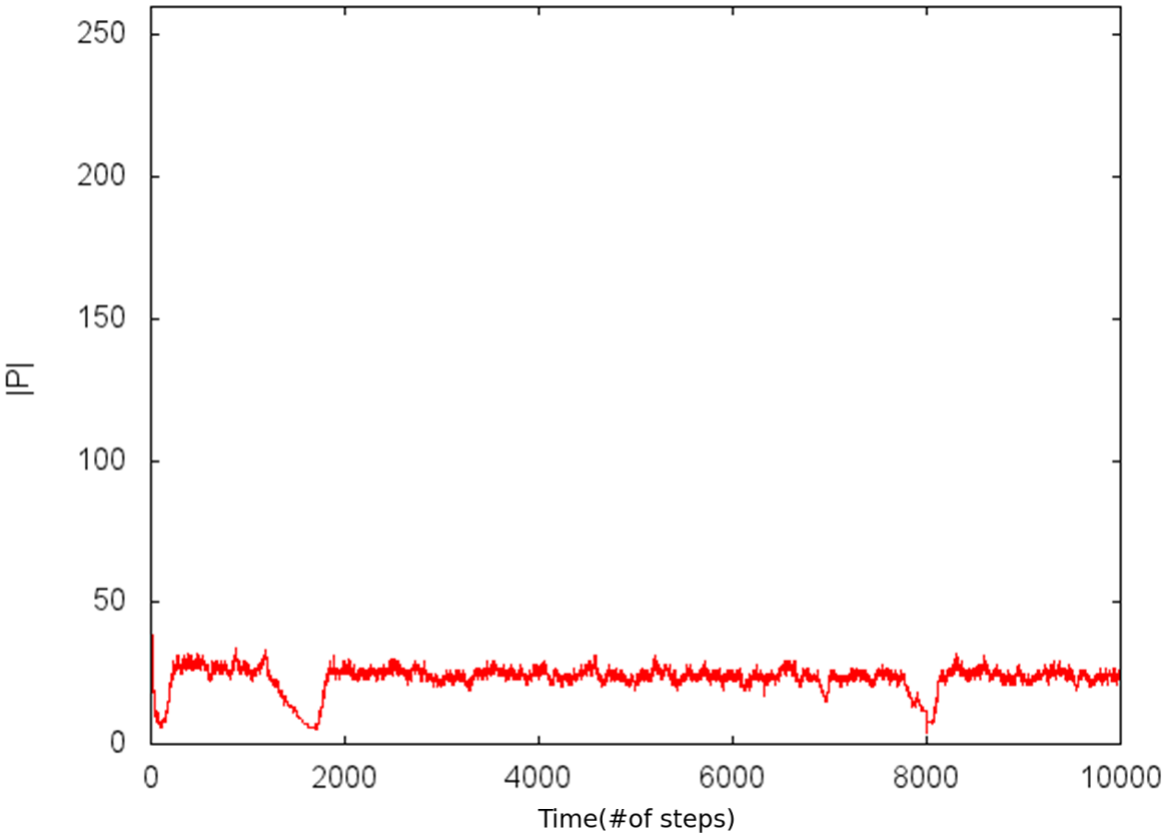
Paretian set cardinality in time. With *P*_*transf*_ = 0.3, *r* = 10 y *J* ∈ [−60, 60].

In Fig 5 the cardinality of the Paretian set is plotted in time.

Using this example the relevant indicators were,

$$\begin{array}{l} \left(2,3,3,0\right),\left(1,1,1,0\right),\left(0,0,3,1\right),\left(3,0,1,1\right),\left(3,0,2,2\right),\left(2,0,3,3\right),\left(1,0,3,3\right),\\ \left(1,0,2,2\right),\left(3,1,1,2\right),\left(1,0,2,1\right),\left(2,0,3,1\right),\left(2,0,2,1\right),\left(1,0,1,0\right),\left(0,0,2,0\right),\;.\\ \left(1,1,3,0\right),\left(1,0,2,3\right). \end{array}$$ (10)

The spatial location of the relevant set, for the example of Cuernavaca, suggests that the indicators that propound sustainable development are strongly related to environmental and social dimensions, with strong institutional components too. This result suggests paying greater attention to indicators with social features to promote a sustainable situation. Since 2006 Mexico has been embedded in a severe humanitarian and social crisis brought by a military security strategy [21, 22]. For that reason democracy, social welfare programs and infrastructural developments have been widely proposed [23]. For future work, application of the model will need to identify indicators that can be measured in real situations and that could be used to define policies to solve actual problems.

**Fig 6:**
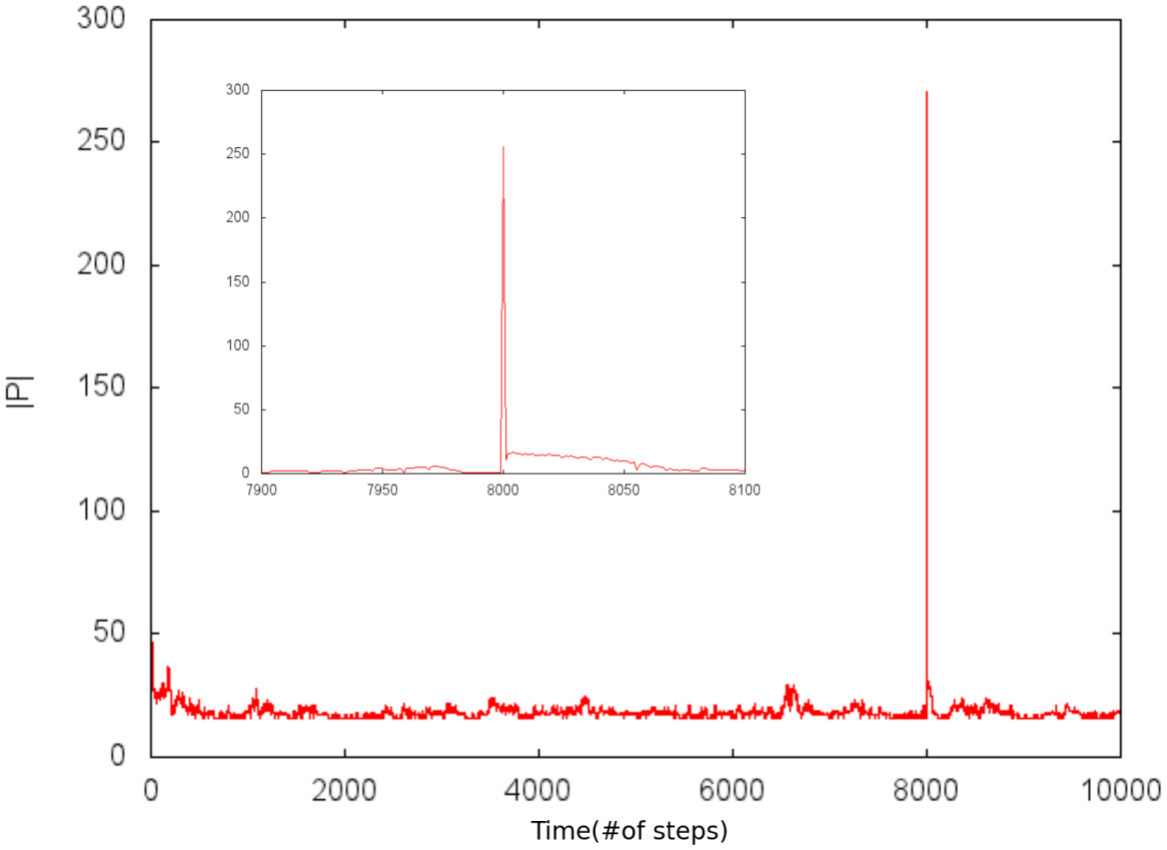
Example of a perturbation. Eliminates all the elements of the Paretian set excepting one showing the return to the original state.

During a stable configuration in which a perturbation in the Paretian set can be applied, we removed all of the fortitude from all indicators, resulting in an inability of the system to recover and subsequently, its death. By reducing the number of indicators being removed from the Paretian set, the system has the ability to recover to the original state (Fig 6), so the removed indicators regain fortitude, returning to the previous Paretian set. Accordingly, this shows resilience. We found that if the size of the Paretian set is more than 5% of the complete set of indicators, then the configuration exhibits this kind of resilience. From our point of view when the Paretian set has many indicators the fortitude is spread between them, so that when we remove some indicators from the set the neighbors eventually transfer fortitude and the system recovers. On the other hand, when the Paretian set is small the fortitude is concentrated in a few influenced indicators, which if removed produces devastating consequences for the system. Thus resilience occurs when the competitive interaction of the indicator alone is very weak, so the fortitude starts to grow and it reaches threshold *U*_*transf*_ to transfer fortitude to a neighbor and eventually recover the previous configuration.

## Conclusions

One important challenge for our society is finding pathways towards sustainability. In order to define pathways that are able to be monitored, decision makers require reliable sets of sustainability indicators, in particular, because there are many sustainability indicators from which to choose.

In this work, we modeled sustainability indicators as agents competing for fortitude to adequately represent a system in a specific context. In our model we incorporated to the Tangled Nature framework the interactions between agents, indicators, based on an expansion of space dimensions of sustainability to represent a system with economics, social, environmental and institutional dimensions. Because the size of our model is small we introduced a fluctuation for agents in zero interaction which we called zero field fluctuation.

The model is capable of simulating different situations or contexts according to the values chosen for *J* ^0^, this interaction with the cooperation-competition dynamics creates an entangled process where self-organization emerges. By contrast, in an ecological system the mechanism of self-organization is independent of the values of *J* ^0^. For the concentration of fortitude, this dynamic is characterized by a group of indicators, establishing relevant indicators for a sustainable system. By determining Pareto sets we define a process of identifying a set of indicators surmised to monitor the pathway to sustainability. We do not refer to sensitivity analysis of the outcomes; rather, our paper is based in the resilient property of the Paretian set. The agents used in this paper are an abstract representation of indicators, so it is necessary to give a relation of the agents used in the model with specific sustainability indicators. By selecting only specific *J* ^0^ we are able to model different contexts form towns, cities, countries, continents or the entire planet.

This model presents metastable states with well defined indicator set showing resilience, however the study of phase transition is a pending task planned for future studies.

## Supporting Information

S1 Text Survey process with experts to represent a specific scenario *J* ^0^. (TXT)
